# Pathogenic mechanisms and potential therapeutic targets for Parkinson disease revealed by bioinformatic analysis of necroptosis and immune cell infiltration

**DOI:** 10.1097/MD.0000000000035311

**Published:** 2023-09-29

**Authors:** Zilong Lin, Jiana Zhang, Runa Wu, Guanmei Chen, Jieying Peng, Renai Li, Shengqiang Chen

**Affiliations:** a Clinical Medicine Program of the Second Clinical College, Guangzhou Medical University, Guangzhou, China; b Neurology Institute, The Second Affiliated Hospital of Guangzhou Medical University, Guangzhou Medical University, Guangzhou, China.

**Keywords:** bioinformatics analysis, immune infiltration, necrotizing apoptosis, neurodegenerative disease, Parkinson disease

## Abstract

Parkinson disease (PD) is an age-dependent neurodegenerative disease with very high prevalence by age 80 years. Necroptosis is a newly identified form of programmed cell death implicated in neurodegenerative diseases, but has not yet been conclusively associated with PD. This study examined the contributions of necroptosis to PD using bioinformatics analysis. Datasets GSE26927, GSE49036, and GSE54536 from the gene expression omnibus database were analyzed for differentially expressed genes (DEGs). These DEGs were then subjected to gene ontology and Kyoto encyclopedia of genes and genomes (KEGG) pathway enrichment analysis to identify associated functions and signaling mechanisms. Necroptosis-related differentially expressed genes (NRDEGs) were then identified by the overlap of DEGs and the necroptosis gene set hsa04217. The STRING database and Cytoscape software were then used to build and visualize a protein–protein interaction network and identify hubs and key functional modules among NRDEGs. In addition, immune cell type abundance was analyzed based on DEGs using ImmuCellAI. The identified DEGs, KEGG pathway enrichment terms, and protein–protein interaction network structures of NRDEGs were validated using an independent dataset (GSE54536). The necroptosis pathway was significantly enriched and activated in PD samples. Thirteen NRDEGs were identified in the GSE26927 and GSE49036 datasets, including receptor interacting serine/threonine kinase 1, CASP8 and FADD like apoptosis regulator, TNFRSF1A associated via death domain, and interleukin 1 beta, of which 6 were validated in the GSE54536 dataset. According to gene ontology and KEGG analyses, these NRDEGs are involved in necroptosis-related processes, apoptosis, B cell receptor signaling pathways, and NOD-like receptor signaling pathways. Analysis of DEGs also revealed significant increases in CD8 + T cell and Tex cell infiltration and significant decreases in B cell and T gamma delta cell infiltration within the PD brain. Necroptosis pathways are active in PD and associated with immune cell infiltration. The factors controlling necroptotic signaling and immune infiltration identified in this study may be valuable diagnostic markers and therapeutic targets for PD.

## 1. Introduction

Parkinson disease (PD) is an age-dependent neurodegenerative disorder^[1]^ afflicting approximately 1% of the global population by age 65 years^[2]^ and reaching 4% by age 80.^[3]^ The main symptoms of PD are motor bradykinesia, resting tremor, rigidity, and postural instability,^[4]^ all of which become more severe and treatment intractable with disease progression. Further, late-stage disease may be associated with various non-motor impairments, including dementia. The pathological hallmarks of PD include progressive loss of dopaminergic neurons in the midbrain substantia nigra pars compacta (SNpc) and the appearance of Lewy bodies, intracellular inclusion bodies containing α-synuclein and other synaptic proteins.^[5]^ A complex interplay among environmental and genetic factors determines PD risk,^[2,6]^ and the early pathogenic processes initiating PD onset are still unclear. In contrast, neurodegeneration in PD is strongly associated with oxidative stress, neuroinflammation, and programmed cell death, suggesting that inventions targeting these processes may slow disease progression. However, currently available treatments only target symptoms, such as motor instability, and cannot inhibit disease progression or reverse PD-related damage.^[7]^ Hence, a more complete understanding of PD etiology and pathogenesis is essential for improved risk assessment, diagnosis, and treatment to improve clinical outcome.

Necrotizing apoptosis is a newly discovered form of programmed cell death with hallmark features of necrosis and apoptosis,^[8]^ including loss of membrane integrity, organelle swelling, cell lysis, leakage of intracellular components, and initiation of death receptor signaling pathways.^[8,9]^ In the classical necroptotic pathway,^[9]^ death receptor ligands such as tumor necrosis factor (TNF)-α and interferon-γ activate receptor-interacting protein kinases 1 and 3 (RIPK1 and RIPK3) and mixed-spectrum kinase structural domain-like (MLKL) pseudokinase. Activated RIPK3 binds and activates the downstream actuator MLKL via phosphorylation at serine 232. This RIPK3-mediated phosphorylation of MLKL then promotes the formation of membrane disruption pores, which cause rapid plasma membrane rupture. As a result, intracellular contents are released that act as tissue damage signals and subsequently induce an inflammatory response.

Interest is growing in the potential contributions of necroptosis to idiopathic neurodegenerative diseases (INDs) such as Alzheimer disease (AD), amyotrophic lateral sclerosis, and multiple sclerosis, as well as PD^[10]^ based on studies identifying activation of necroptosis effectors in animal models and human patients. For instance, RIPK1 recruitment was observed in TNF-α-stimulated AD model mice, which resulted in RIPK1 self-oligomerization and activation of the downstream RIPK1/RIPK3/MLKL cascade.^[11]^ Pathological samples from superoxide dismutase 1, soluble (G93A) transgenic mice and human amyotrophic lateral sclerosis patients also provided evidence for RIPK1-mediated axonal degeneration through the promotion of neuroinflammation and necroptosis.^[12]^ It was similarly reported that RIPK1 expression was upregulated in multiple sclerosis brain samples and that the inhibition of RIPK1 could diminish disease progression and inhibit deleterious pro-inflammatory signaling among astrocytes and microglia.^[13]^ Necroptosis may also contribute to PD development,^[14–17]^ but this has not been confirmed in human tissue samples.

Identification of differentially expressed genes (DEGs) in diseased brain tissue and subsequent bioinformatics analysis of associated pathways and functions may reveal previously unknown pathogenic mechanisms and novel treatment targets. Indeed, RNA sequencing has already revealed numerous DEGs associated with PD pathogenesis,^[18]^ while plasmapheresis-based proteomics holds great promise for identifying additional pathogenic mechanisms and treatment targets.^[19]^ In the present study, we downloaded PD-related datasets from the publicly available gene expression omnibus (GEO) database, screened for DEGs, examined the role of necroptotic apoptotic pathways in the development of PD by enrichment analysis, and identified necroptosis-related DEGs (NRDEGs) and their key functional networks. We then validated our findings with independent datasets from blood sources. These findings strongly implicate necroptosis in the progression of PD and identifies numerous potential molecular targets for diagnosis and treatment.

## 2. Materials and methods

**Figure 1.**
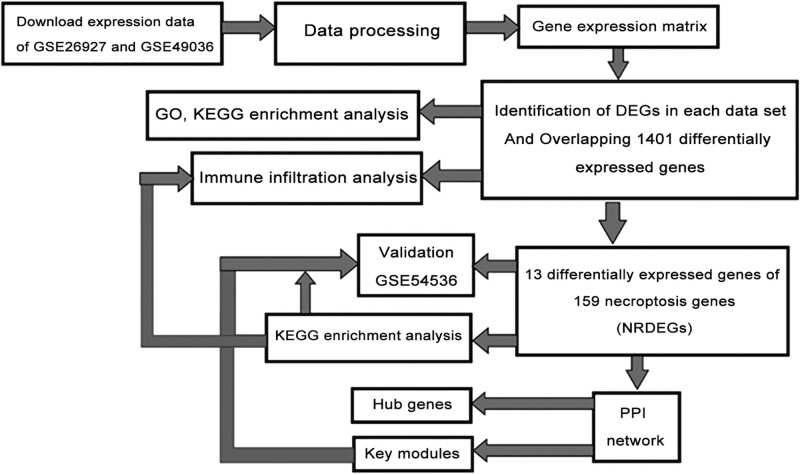
Flow chart of the analysis strategy for identifying necroptosis-related genes contributing to Parkinson disease pathogenesis.

Our primary goal was to identify necroptosis-associated genes and proteins differentially expressed in PD brain to reveal potential pathogenic mechanisms and therapeutic targets. The work flow is illustrated in Figure 1.

### 2.1. Data source

We searched the NCBI-GEO database (http://www.NCBI.nlm.nih.gov/GEO/) using “PD” as a keyword to identify experimental studies that screened for genetic differences between PD patients and healthy individuals. Additional inclusion criteria included the following: the study was approved by the ethics committee; raw genetic data were available; the study was performed on the substantia nigra of the brain or the blood of PD patients; the raw gene microarray was of high quality (percentage of gene matches >90%). The GSE26927^[20]^ and GSE49036^[21]^ nigrostriatal datasets of PD patients were selected. The GSE26927 dataset contains expression profiles of 12 PD brain tissue samples and 8 healthy brain tissue samples obtained using the GPL6255 Illumina humanRef-8 v2.0 expression beadchip. The GSE49036 dataset consists of expression data from 20 PD brain tissue samples and 8 healthy brain tissue samples obtained using the GPL570 (HG-U133_Plus_2) Affymetrix Human Genome U133 Plus 2.0 Array. Additionally, the GSE54536^[22]^ dataset including expression profiles of 5 peripheral blood samples from PD patients and 5 from healthy controls obtained using the GPL10558 Illumina HumanHT-12 V4.0 expression beadchip was analyzed for external validation. As datasets used in the study were derived from GEO data, ethical approval and informed consent requirements were waived by the institution research ethics committee.

### 2.2. Selection of differentially expressed genes

NetworkAnalyst is an online visual analysis platform for gene expression profiling and meta-analysis that integrates advanced statistical methods and innovative data visualization for efficient data comparison, biological interpretation, and hypothesis generation.^[23,24]^ In this study, each dataset was processed to screen for DEGs using NetworkAnalyst. We performed data quality checks, data filtering, and normalization of the 2 screened PD patient nigra datasets, and then performed DEG selection based on *P* < .05 and | log FC | > 0.1 by Limma statistical methods. Then DEGs common to both datasets were identified using a Venn diagram. Volcano maps and Venn diagrams were produced using the Bioinformatics “bioinformatics” tool (http://www.bioinformatics.com.cn).

### 2.3. Functional enrichment analysis

The functions and associated pathways of these DEGs were then identified using the Kyoto encyclopedia of genes and genomes (KEGG) orthography-based annotation system (KOBAS), a web server for gene/protein function annotation (annotation module) and function set enrichment (enrichment module).^[25]^ A *P* value < .05 was considered statistically significant. The results of KOBAS analysis, including both gene ontogeny (GO) and KEGG annotations, were visualized using the Bioinformatics “bioinformatics” tool (http://www.bioinformatics.com.cn).

### 2.4. Screening and pathway analysis of necroptosis-related DEGs (NRDEGs)

Genes differentially expressed in PD were then examined for enrichment of necroptosis-related genes according to GO and KEGG. To further explore the role of necroptosis in the development of PD, the M24779.gmt gene set from the gene set enrichment analysis database (http://www.gsea-msigdb.org/gsea/index.jsp) was screened for necrotrophic/apoptotic genes. Moreover, gene maps associated with necroptosis were collected from the KEGG pathway database (https://www.genome.jp/dbget-bin/www_bget?pathway+hsa04217). Detailed information on these genes is provided in Supplementary material S3, http://links.lww.com/MD/K6. Necroptosis-related (NR) DEGs were identified by a Venn diagram as the overlap of DEGs and the KEGG gene set (hsa04217). In addition, heat maps were used to visualize the expression levels of NRDEGs in both GSE26927 and GSE49036 datasets. The KOBAS server was then used to identify functions and related pathways of NRDEGs outside the necroptosis pathway. A *P* value < .05 was considered statistically significant. Venn diagram and heat maps were produced and KEGG analysis results visualized using Bioinformatics “bioinformatics” tools (http://www.bioinformatics.com.cn).

### 2.5. Protein–protein interaction (PPI) networks and identification of hub genes and key modules

Complex cellular functions are achieved through protein interactions. For identification of interacting proteins, a PPI network of NRDEGs was constructed using the STRING database (https://string-db.org/cgi/input.pl)^[26]^ with default parameters (with the parameter of a factor = 0.4). and optimized using Cytoscape (version 3.7.0).^[27]^ The Cytoscape plugin CytoHubba MCC algorithm was used to identify hub genes, defined as those with the top 10 highest interaction scores, while the MCODE plugin was used to filter out key modules in the PPI network.^[28,29]^

### 2.6. Immune infiltration analysis

Immune Cell Abundance Identifier (ImmuCellAI) (http://bioinfo.life.hust.edu.cn/web/ImmuCellAI)^[30]^ is an online tool that uses gene expression data to estimate the abundance of 24 immune cell types (including 18 T cell subtypes and 6 other immune cells [B cells, natural killer cells, monocytes, macrophages, neutrophils, and dendritic cells]) in sample tissue. Common DEGs and NRDEGs were significantly enriched in multiple immune-related pathways according to the KEGG enrichment outcomes. The abundance of 24 immune cell types in the GSE26927 dataset was analyzed by ImmuCellAI. An infiltration score was defined for each infiltrating immune cell type.

### 2.7. Validation of necroptosis in PD using the GSE54536 gene set

The NRDEGs identified in the GSE26927 and GSE49036 datasets were further validated by examining the GSE54536 dataset using the Limma statistical method in GEO2R online analysis. Briefly, DEGs were screened at *P* < .05, | log FC | >0.1. KEGG pathway enrichment analysis was conducted and visualized using KOBAS^[25]^ and the Bioinformatics “bioinformatics” tool (http://www.bioinformatics.com.cn). A *P* < .05 was considered statistically significant for all tests. A heat map of NRDEGs was produced using the Bioinformatics “bioinformatics” tool (http://w.bioinformatics.com.cn). In addition, a PPI network was constructed from GSE54536 NRDEGs using STRING (https://string-db.org/cgi/input.pl)^[26]^ and then plotted and optimized using Cytoscape (version 3.7.0).^[27]^ The key modules in the PPI network were filtered separately using the Cytoscape plugin MCODE.^[28,29]^

## 3. Results

### 3.1. Identification of DEGs in the SNpc of PD patients

**Figure 2.**
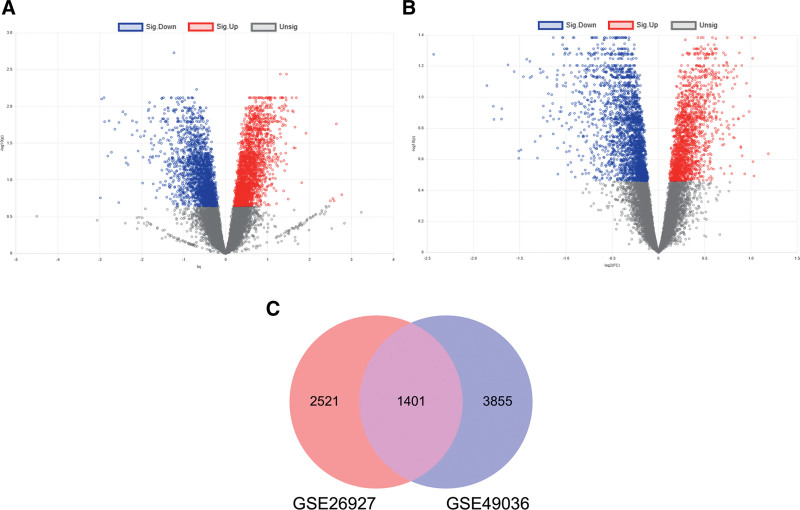
Screening for differentially expressed genes (DEGs) in the SNpc of PD patients. (A) Volcano map showing the DEGs of the NCBI-GEO database dataset GSE26927 (PD vs controls). (B) Volcano map showing the DEGs of dataset GSE49036. (C) Venn diagram showing overlapping DEGs from GSE26927 and GSE49036 datasets. GEO = gene expression omnibus, PD = Parkinson disease, SNpc = substantia nigra pars compacta.

Transcriptomic analysis is an efficient method to describe large-scale changes in gene expression during disease onset and development. A total of 3922 DEGs were identified between PD patients and healthy individuals in the GSE26927 dataset, of which 1840 were upregulated and 2082 downregulated. In the GSE49036 dataset, a total of 6970 DEGs were identified, among which 4029 were upregulated and 2941 were downregulated. These DEGs are shown in the volcano plots of Figure 2A and B, respectively, and 1401 DEGs common to both are shown in the Venn diagram of Figure 2C.

### 3.2. Functional analysis and mechanism exploration

**Figure 3.**
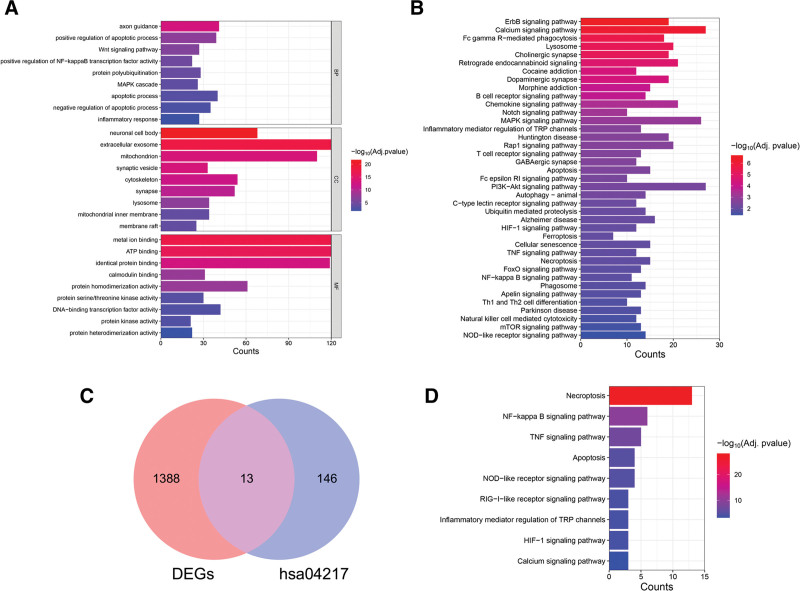
Functions of PD-associated DEGs. (A) Enriched gene ontology (GO) terms for DEGs from GSE26927 and GSE49036 datasets. (B) Enriched KEGG pathway terms for DEGs from GSE26927 and GSE49036 datasets. (C) Venn diagram showing overlap between DEGs and KEGG necroptosis gene set hsa04217 (NRDEGs). (D) Enriched KEGG pathway terms for NRDEGs. BP = biological process; CC = cellular component; KEGG = Kyoto encyclopedia of genes and genomes, MF = molecular function, NRDEGs = necroptosis-related differentially expressed genes, PD = Parkinson disease.

The KOBAS application identified 908 GO terms and 140 KEGG signaling pathways (Supplementary Material S1, http://links.lww.com/MD/K4 and S2, http://links.lww.com/MD/K5) significantly related to these 1401 common DEGs. The top GO biological process terms were “positive regulation of apoptotic process,” “Wnt signaling pathway,” “positive regulation of NF-kappaB transcription factor activity,” “apoptotic process,” “inflammatory response,” and “negative regulation of apoptotic process,” the top GO cellular components terms were “neuronal cell body,” “extracellular exosome,” “mitochondrion,” “membrane raft,” and “lysosome,” and the top GO molecular function terms were “metal ion binding,” “ATP binding,” “identical protein binding,” “protein homodimerization activity,” and “DNA-binding transcription factor activity.” These common DEGs were also enriched in KEGG pathway terms “Dopaminergic synapse,” “Apoptosis,” “Autophagy–animal,” “Ferroptosis,” “Cellular senescence,” “Necroptosis,” “TNF signaling pathway,” “B cell receptor signaling pathway,” “T cell receptor signaling pathway,” “Th1 and Th2 cell differentiation,” “Natural killer cell mediated cytotoxicity,” “mTOR signaling pathway” and “NOD-like receptor signaling pathway” (Fig. 3A and B).

### 3.3. Screening and functional annotation of necroptosis-related DEGs (NRDEGs)

The set of common DEGs was significantly enriched in genes related to the necroptotic pathway. First, the 8 necroptosis-related genes in the gene set enrichment analysis database M24779.gmt gene set completely overlapped with the 159 genes associated with necroptosis from the KEGG pathway database (gene set hsa04217). Venn diagram analysis of the overlap with common DEGs from GSE26927 and GSE49036 datasets revealed 13 NRDEGs (Fig. 3C). These NRDEGs were also significantly enriched in genes of the KEGG pathways “NF-kappa B signaling pathway,” “TNF signaling pathway,” “NOD-like receptor signaling pathway,” “RIG-I-like receptor signaling pathway” and “other immune-related pathways except in necrotizing apoptosis” (Fig. 3D).

### 3.4. Network analysis of NRDEGs and identification of hub genes

**Figure 4.**
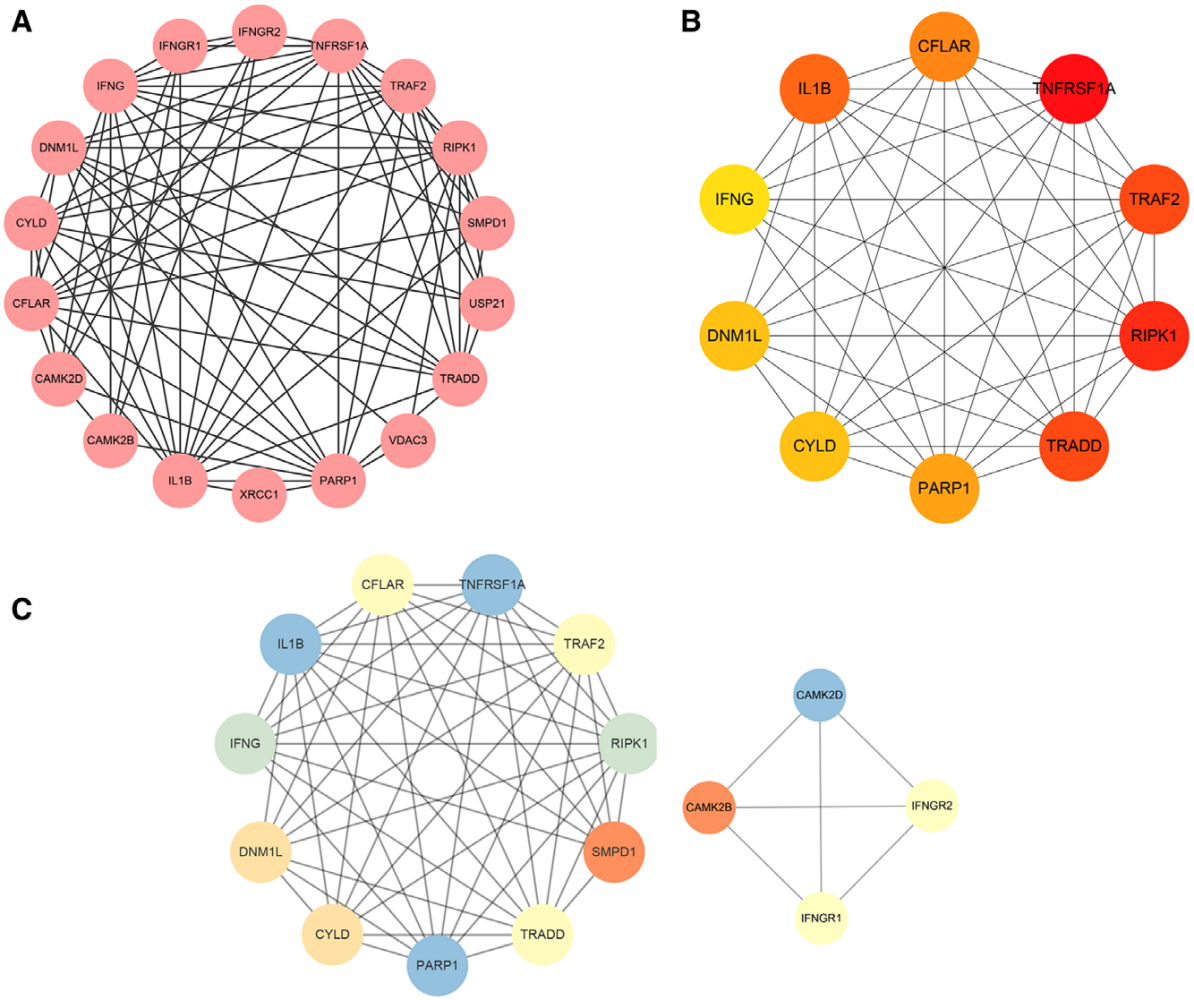
Protein–protein interaction (PPI) network and key modules constructed from NRDEGs by STRING and Cytoscape. (A) There are 79 edges and 18 nodes in the PPI network. (B) The top 10 hub genes as identified by CytoHubba. (C) Clustering analysis of the molecular complex detection network. Low degrees are shown by bright colors. NRDEGs = necroptosis-related differentially expressed genes.

To explore potential key molecular targets of NRDEGs involved in PD pathogenesis, we constructed a PPI network of NRDEGs using the STRING database (Fig. 4A) and analyzed the results for nodes using Cytoscape. The top 10 hub genes identified by the CytoHubba MCC algorithm were CASP8 and FADD like apoptosis regulator (CFLAR), TNF receptor superfamily member 1A, TNF receptor associated factor 2, RIPK1, TNFRSF1A associated via death domain (TRADD), poly (ADP-ribose) polymerase 1, CYLD lysine 63 deubiquitinase (CYLD), dynamin 1 like (DNM1L), interferon gamma, and interleukin 1 beta (IL1B) (Fig. 4B). According to the MCODE plugin, a module consisting of 11 nodes and 50 edges was identified as an important cluster in the PPI network (Fig. 4C). Based on these findings, we suggest that the NRDEGs CFLAR, RIPK1, TRADD, poly (ADP-ribose) polymerase 1, CYLD, DNM1L, and IL1B may have strong influences on PD pathogenesis and thus could be potential targets for treatment.

### 3.5. Immune infiltration analysis

**Figure 5.**
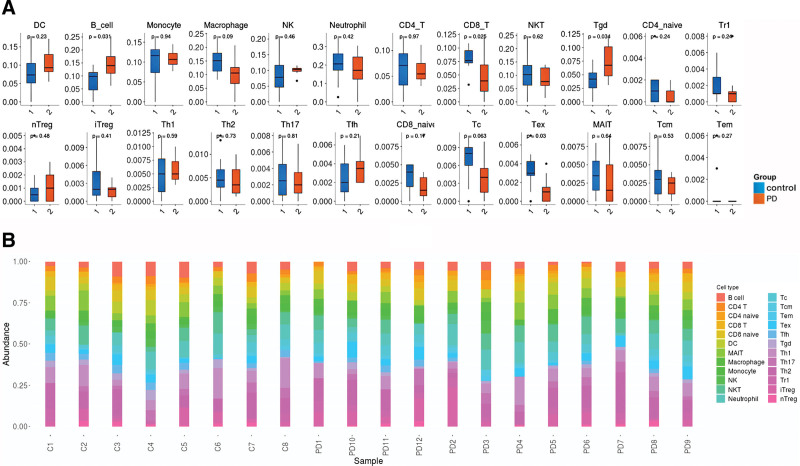
Differences in immune cell infiltration of the SNpc between PD patients and controls (dataset GSE26927). (A) Box plots of specific immune cell frequencies (% of the total number) in brain samples from PD patients and healthy controls. (B) Stacked bar graphs of immune cell types. PD = Parkinson disease, SNpc = substantia nigra pars compacta.

The identified DEGs and NRDEGs were also highly enriched in GO and KEGG terms related to immune system function. Therefore, we assessed the associations between DEGs of the GSE26927 gene set and infiltrating immune cell phenotypes using ImmuCellAI and found that 4 cell types among the 24 differed significantly in the substantia nigra of PD patients compared to healthy controls (Fig. 5A and B). Of these 4 cell types, CD8 T cells and exhausted CD8 T cell (Tex cells) were enriched in the PD group while B cells and T gamma delta cell (Tgd cells) were enriched in the healthy group.

### 3.6. Validation of NRDEGs

**Figure 6.**
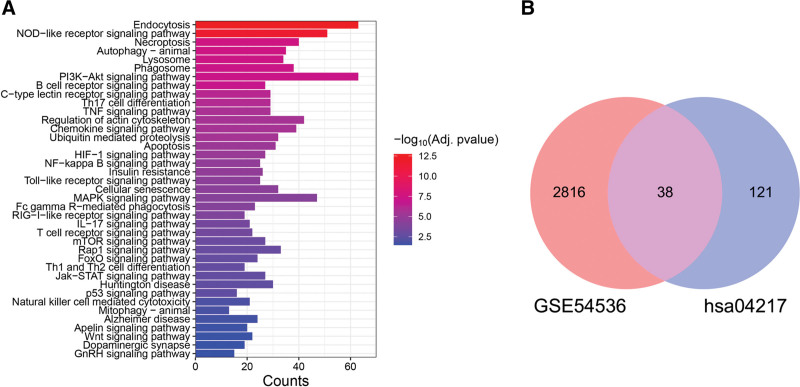
Functional enrichment analysis of the verification dataset (GSE54536). (A) Enriched Kyoto encyclopedia of genes and genomes (KEGG) terms for the differentially expressed genes (DEGs). (B) Venn diagram showing overlapping genes (NRDEGs) between DEGs in GSE54536 and the necroptosis gene set hsa04217.

**Figure 7.**
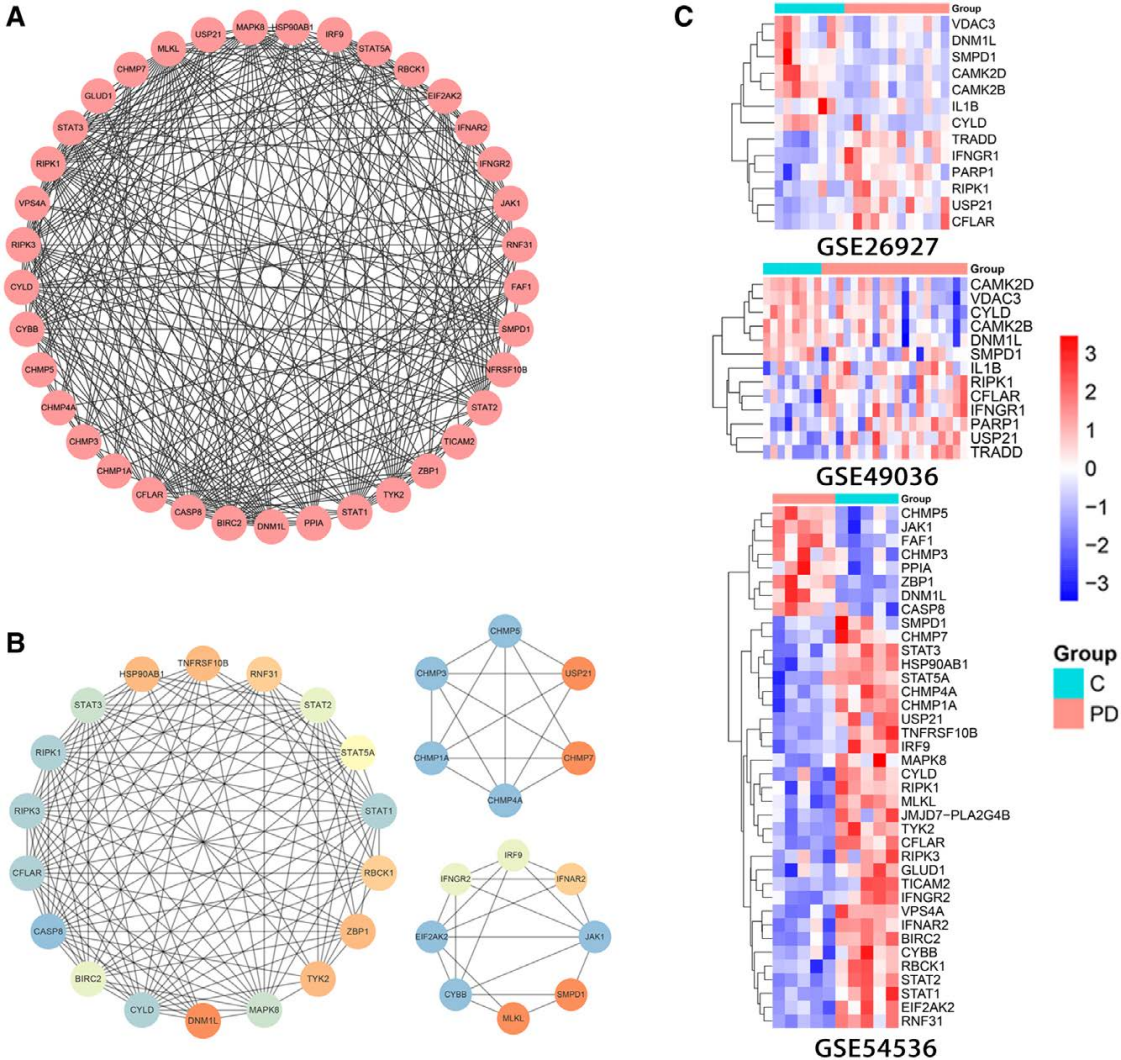
The PPI network and key modules for the validation dataset. (A) PPI network key modules constructed from NRDEGs in dataset GSE54536 by STRING and Cytoscape. There are 311 edges and 38 nodes in the PPI network. (B) Key modules constructed from NRDEGs in GSE54536 by STRING and Cytoscape. Clustering analysis of the molecular complex detection network with low degrees shown as bright colors. (C) Cluster heat maps of NRDEGs in GSE26927, GSE49036, and GSE54536. NRDEGs = necroptosis-related differentially expressed genes, PPI = protein–protein interaction.

The DEGs between PD and healthy control also related to necroptosis (NRDEGs) were then validated using dataset GSE54536. In total, 3108 DEGs were extracted, of which 2472 were upregulated and 634 were downregulated. These DEGs were significantly enriched in genes of the KEGG pathways “Necroptosis,” “Cellular senescence,” “Apoptosis,” “Autophagy—animal,” “B cell receptor signaling pathway,” “Th17 cell differentiation,” “NOD-like receptor signaling pathway,” and “Toll-like receptor signaling pathway” (Fig. 6A and Supplementary Material S4, http://links.lww.com/MD/K7). In addition, we extracted 38 NRDEGs (Fig. 6B), constructed PPI networks using the STRING database, visualized these networks using Cytoscape (version 3.7.0), and filtered the key modules in the PPI networks using the Mcode plug-in (Fig. 7A and B). Six of the 13 NRDEGs identified in datasets GSE26927 and GSE549036 were also identified in GSE54536, including RIPK1, CYLD, CFLAR, sphingomyelin phosphodiesterase 1, DNM1L, and ubiquitin specific peptidase 21. In general, the results were relatively consistent between the discovery and validation sets.

## 4. Discussion

There is compelling evidence that necroptosis contributes to the pathophysiology of INDs, including PD. However, this evidence was obtained mainly from mouse models and human cell models, such as the human neuroblastoma cell line SH-SY5Y, and has not been confirmed in human brain samples. Here, we confirmed the differential expression of necroptosis-related genes in brain samples from PD patients compared to healthy controls by bioinformatics analyses. In brief, the evidence for necroptosis in PD can be summarized as follows: differential expression of RIPK1, TRADD, and 32 other necroptosis-related genes in brain samples from PD patients; enrichment of necroptosis-related GO terms and KEGG pathways among DEGs between patients and controls; and efficient construction of a PPI network from NRDEGs. In addition, enrichment analysis revealed that NRDEGs were involved in inflammatory response pathways such as the TNF, NOD-like receptor, Toll-like receptor, and IL-17 signaling pathways. We also found significant differences in infiltrating immune cell types between PD and healthy brain samples using ImmuCellAI, including enrichment of CD8 T cells and exhausted cells, and reduced B cells and gamma delta cells in PD brains compared to healthy brains. To the best of our knowledge, this is the first bioinformatics study reporting necrotizing apoptosis in human PD brain, strongly suggesting that this form of programmed cell death contributes to PD-related neurodegeneration and neurological deficits.

In this study, a large number of DEGs were identified from GEO datasets comparing SNpc expression profiles between PD patients and controls only by using a relatively low differential expression threshold (| log FC | > 0.1). However, relatively small changes in gene expression level can have substantial effects on neurological function. Further, genes associated with cell death pathways are expected to be upregulated only in a small fraction of neurons at a given time, and our aim was to identify PD-related DEGs that are also associated with necroptosis. This strategy was also used in previous studies on DEGs associated with PD, such as |log2 FC| > 0.2 by Zhao and colleagues and | log2FC | > 0.15 by Hu and colleagues.

Progressive neuronal loss in the SNpc is the cardinal pathological felyature of PD.^[31]^ Pathology begins in SNpc terminals within the striatum and progresses in a retrograde manner to the cell somata.^[32–35]^ Emerging evidence suggests a critical role for programmed cell death pathways in this retrograde neurodegeneration, including intrinsic and extrinsic apoptosis, necroptosis, ferroptosis, parthanatos, and mitochondrial permeability transition-driven necrosis.^[36]^ For instance, it was reported that phosphorylation of the anti-apoptotic protein BCL2 prevented the death of dopaminergic neurons in an animal model of PD by balancing apoptotic and autophagic pathway signaling.^[37–39]^ Further, several studies have suggested that iron-dependent cell death (ferroptosis) contributes to the development of PD.^[40–42]^ In accord with the current findings, PD model animal studies have also implicated necroptosis in the death of dopaminergic neurons.^[17,43]^ KEGG analysis of DEGs suggested that both necroptotic and canonical apoptotic signaling pathways are activate in the SNpc of human PD patients. In addition, construction of a PPI network identified necroptosis-associated proteins as network hubs and components of key network modules, including RIPKs and TRADD. Post-translational modifications of RIPK complex 1, including ubiquitination, deubiquitination, and phosphorylation, are critical determinants of cell death, mode of cell death, survival, and cellular inflammatory responses. Accumulating evidence suggests that activated (phosphorylated) RIPK1, RIPK3, and MLKL form protein aggregates and amyloidogenic fibers that promote necroptosis and neurodegeneration.^[44]^ Another pathological hallmark of PD is the appearance of intracellular inclusions containing α-synuclein, termed Lewy bodies,^[5]^ and these inclusions appear to trigger apoptosis and necroptosis. Tumor necrosis factor receptor TRADD acts as a linker molecule facilitating multiprotein complex formation in various signaling pathways controlling cell survival, proliferation, differentiation, apoptosis, necroptosis, and inflammation. For example, TRADD binds to TNFR1 in the presence and absence of RIPK1, and also interacts with TNF receptor associated factor 2 to form a complex that in turn activates nuclear factor-κB (NF-κB) and jun N-terminal kinase mitogen-activated protein kinase pathways to generate anti-apoptotic and pro-inflammatory responses. In addition, when NF-κB activation is inhibited, TRADD binds FAD to initiate the apoptotic cascade. Furthermore, it has been shown that TRADD is involved in the formation of the RIPK1–TRADD–FADD–cysteine aspartase (caspase)-8 complex, which activates necroptosis. Furthermore, TRADD binds RIPK3 and promotes RIPK3 activation, which then initiates necroptotic signaling in response to TNF-αstimulation. It was reported that RIPK1 activates RIPK3 via RIP isoform interaction motif-dependent PPIs. Activation of RIPK3 then induces MLKL phosphorylation, which subsequently triggers necroptosis. Other members of the TNF receptor superfamily (TNFRSF), such as DR3, TRAILR1 (DR4), TRAILR1 (DR5), DR6, and p75 NTR, also recruit TRADD upon ligand activation, which then propagates to downstream cell death signaling pathways.^[45–48]^ Research on TRADD functions in INDs have focused primarily on AD,^[48,49]^ and to our best knowledge, the current study is the first to demonstrate the involvement of TRADD in PD-related necroptosis, although this requires further confirmation in PD mouse models. Genes associated with Toll-like receptor signaling were also differentially expressed in PD brain, and these Toll-like receptor pathways are known to promote exogenous apoptosis and necroptosis.^[50]^ Thus, various programmed cell death pathways may collectively contribute to IND pathogenesis, further suggesting that signaling components of these pathways are promising therapeutic targets.

In contrast, the NRDEGs CFLAR can block apoptotic signaling. It was reported that Bclaf1 suppresses apoptosis by promoting the transcription of CFLAR, a cystatin 8 antagonist downstream of NF-κB activation, suggesting that modulation of CFLAR expression influences necroptosis.^[51]^ At present, CFLAR has not been studied extensively in INDs, although studies on CFLAR functions in other diseases may provide clues to roles in IND pathogenesis.

Plasma membrane rupture during necroptosis causes the release of intracellular contents into the extracellular space,^[9]^ which stimulates resident immune cells to release interleukin (IL)-1β and other pro-inflammatory factors. The release of IL-1β from microglia is strongly implicated in AD-associated neuroinflammation, and disruption of this proinflammatory signaling pathway by blocking CD33 has been reported to reduce amyloid β accumulation and neuroinflammation in AD model mice.^[52]^ KEGG analysis identified several immune-related pathways in PD samples, including “B cell-mediated immunity,” “NOD-like receptor signaling,” “Th17 cell differentiation,” “T cell differentiation involved in the immune response,” and “Th2 cell differentiation.” These findings were further confirmed by subsequent immune infiltration analysis.

ImmuCellAI results suggested that infiltrating CD8 T cells and Tex cells were significantly enriched in the SNpc of PD patients, whereas infiltrating B cells and Tgd cells were reduced compared to healthy brain. These findings are consistent with previous reports that CD8 T cells are elevated in diagnosed PD cases and that density is positively correlated with neuronal death.^[53]^ In addition, it was reported that “naïve” T cells, CD4 + T cells, and CD19 + B cells were reduced while cytotoxic CD8 + T cells were elevated in PD brain. The depletion of naive T cells (CD95−) was associated with weakened immune protection in the elderly and clonal expansion of CD28-T cells, which may acquire cytotoxicity under certain conditions.^[54]^ The reduced number of Tdg cells is consistent with lower numbers in the peripheral blood of PD patients reported in 1 study^[55]^ and corroborated by bioinformatics analysis of significant differences in PD immune cell fractions.^[56]^ However, contradictory results have also been reported,^[57]^ so further studies are required to confirm Tgd cell changes in PD. A decrease in the number of B cells in PD has also been reported in several previous studies,^[58–61]^ although the mechanisms and effects on disease progression are unclear.

While the current study suggests a strong association between necroptosis and immune cell infiltration in INDs, it should be noted that the relationships between necrotizing apoptosis and immune infiltration are complex. For example, cystathione aspartase acts in necroptosis, while cystathione-8 also contributes to the maintenance and homeostasis of adult T-cell populations. In addition, cystathione-3 contributes to tissue differentiation, regeneration, and neurodevelopment in the absence of apoptotic activity.^[62]^

This study had several limitations. Patient details are not fully available from public databases, so potential confounding factors (such as age, co-morbidities, and postmortem delay) could not be controlled using statistical methods. Also, many of the identified pathways and targets have not been corroborated in PD patients or animal models.

## 5. Conclusion

Our bioinformatics analysis of brain tissue from PD patients strongly implicates necroptosis and associated neurodegenerative pathways in disease progression. More importantly, these finding provided a rich resource for exploring the pathomechanisms of PD and new targets for medical intervention. Future work on the associations and interactions among neuroinflammatory and necroptotic signaling pathways may provide important clues to PD pathogenesis and treatment.

## Acknowledgments

We are grateful for the establishment and sharing of the GEO database.

## Author contributions

**Data curation:** Zilong Lin.

**Methodology:** Zilong Lin.

**Project administration:** Shengqiang Chen.

**Supervision:** Shengqiang Chen.

**Validation:** Zilong Lin.

**Visualization:** Zilong Lin.

**Writing – original draft:** Zilong Lin, Jiana Zhang, Runa Wu, Guanmei Chen, Jieying Peng, Renai Li.

**Writing – review & editing:** Shengqiang Chen.

## Supplementary Material








